# Epigenetic aging and cancer incidence in a German cohort of older adults

**DOI:** 10.1038/s41514-026-00356-y

**Published:** 2026-03-09

**Authors:** Qiming Yin, Joshua Stevenson-Hoare, Bernd Holleczek, Ben Schöttker, Hermann Brenner

**Affiliations:** 1 Division of Clinical Epidemiology of Early Cancer Detection, German Cancer Research Center (DKFZ), Heidelberg, Germany; 2 Medical Faculty Heidelberg, Heidelberg University, Heidelberg, Germany; 3 Saarland Cancer Registry, Saarbrücken, Germany; 4 Network Aging Research, Heidelberg University, Heidelberg, Germany; 5 Cancer Prevention Graduate School, German Cancer Research Center (DKFZ), Heidelberg, Germany

**Keywords:** Biomarkers, Cancer, Oncology, Risk factors

## Abstract

Rising life expectancy and an aging population highlight the importance of cancer control. DNA methylation (DNAm)-based biological age (BA) may provide insights into aging, carcinogenesis, and cancer prevention and care. We estimated five BA metrics among 1916 participants aged 50–75 years at baseline in the German ESTHER cohort, with repeat BA measurements available for 894 participants after 8 years. Multivariable linear regression was used to assess associations between prior cancer and baseline BA, while Cox proportional hazards models and restricted cubic splines evaluated associations of BA levels and trajectories with subsequent cancer risk. A history of malignant tumors was associated with higher baseline PCHannum and PCGrimAge in fully adjusted models. Older BA levels were significantly associated with increased long-term cancer risk, with hazard ratios up to 1.67 (95% CI 1.25–2.24) per standard deviation (SD) increase in PCGrimAge. Except for PCGrimAge, BA trajectories showed monotonic, linear associations with cancer risk, corresponding to a 33% to 37% higher risk per SD increase in slopes of four BA matrices. Accelerated biological aging was consistently associated with increased overall cancer risk, highlighting the potential value of longitudinal BA measures for cancer risk assessment, prevention, and monitoring.

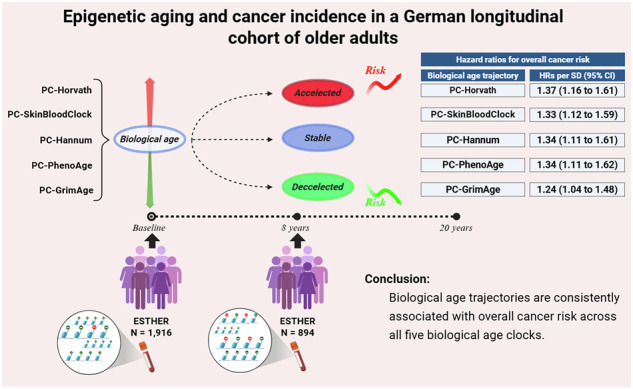

## Introduction

Cancer ranks as a leading cause of death worldwide, accounting for nearly 10 million deaths in 2022^1^. In addition to being an important barrier to increasing life expectancy, cancer is associated with substantial public health burden that varies in degree across cancer types and gender worldwide^2^. Moreover, because the risk of cancer increases exponentially with advancing age^3^, with greater life expectancy, overall cancer burden is expected to rise. These clinical and epidemiological trends underscore the challenges of cancer control in middle-aged and older adults^4^.

Some of the proposed hallmarks of aging, such as genomic instability and epigenetic alteration, also overlap with the hallmarks of cancer^5–9^. However, individuals of the same chronological age (CA) may exhibit different susceptibility to cancer, which is likely reflective of differences in their underlying biological aging processes^10–12^. Understanding the molecular mechanisms underlying the aging process not only informs cancer surveillance but also contributes to the long-term care of cancer survivors, with the ultimate goal of maximizing healthy survival. Biological age (BA) has been proposed as a more accurate indicator of an individual’s global physiological state, capturing variability in aging rates among individuals with the same CA^11,13^. Therefore, BA can be useful to assess health risks in individuals of the same age. DNA methylation (DNAm)-based epigenetic clocks could evaluate BA and are increasingly recognized as robust biomarkers of aging as they have been reported to reflect aging processes^6,9,10,14,15^.

More specifically, there are two generations of DNAm-based BA metrics. The first-generation clocks include Hannum^16^, Horvath^17^, and SkinBloodClock^18^, which derive aging-related DNA methylation signals from specific cytosine-phosphate-guanine (CpG) dinucleotides. In contrast, second-generation clocks like PhenoAge and GrimAge incorporate additional information related to mortality risk and physiological biomarkers^19,20^. More recent, third-generation metrics have been proposed to quantify aging-related biological processes, such as the pace of aging (DunedinPoAm and DunedinPACE)^21,22^. However, many individual CpG sites measured on DNA methylation microarrays are subject to technical variability, which can compromise the reliability of epigenetic clock estimates^23^. To address this issue, principal components (PCs) derived from sets of CpGs, rather than individual CpGs, have been used to construct PC-based versions of epigenetic clocks (PC-clocks), which offer improved robustness, particularly in longitudinal analyses^24^. In the present study, we therefore focused on PC-transformed versions of established first- and second-generation epigenetic clocks, including Horvath, Skin & Blood, Hannum, PhenoAge, and GrimAge.

In this study, we utilized longitudinal DNAm-based BA data, measured eight years apart, from a prospective cohort of community-dwelling adults aged 50 to 75 years in Germany, to evaluate the impact of cancer on changes and trajectories in biological aging. We also aimed to explore the potential value of BA and its trajectory in cancer risk prediction and post-diagnosis surveillance.

## Results

### Study population characteristics

Table 1 shows the baseline sociodemographic characteristics of participants. No significant differences were observed between the total study population and the subgroup with second estimation of DNAm (*p* > 0.05). In both the total study population and the subgroup, mean age was between 61 and 62 years, and a slight majority of participants were women. About three out of four participants were overweight or obese, about half had ever smoked, and mean daily alcohol consumption was ≤ 11 g per day in both samples.

**Table 1:** Baseline characteristics of the study population and the subgroup with repeated DNAm measurements

		Total study population (N = 1916)	Individuals with repeated measurement (N = 894)
Age (years; mean ± SD)		61.6 ± 6.5	61.2 ± 6.3
Sex (N/%)	Female	1062 (55.4)	486 (54.4)
	Male	854 (44.6)	408 (45.6)
Nationality (N/%) ^**a**^	German	1796 (93.7)	840 (94.0)
	Non-German	119 (6.2)	54 (6.0)
Education (N/%) ^**b**^	<= 9 years	1389 (74.1)	622 (71.2)
	10-11 years	269 (14.4)	137 (15.7)
	>= 12 years	216 (11.5)	114 (13.1)
BMI (N/%) ^**c**^	Underweight & Normal range ( < 25.0 kg/m^2^)	483 (25.2)	252 (28.2)
	Overweight (25.0 – 30.0 kg/m^2^)	905 (47.2)	421 (47.1)
	Obese ( > 30.0 kg/m^2^)	525 (27.4)	221 (24.7)
Smoking status (N/%) ^**d**^	Never	953 (49.7)	431 (48.2)
	Former	638 (33.3)	320 (35.8)
	Current	276 (14.4)	115 (12.9)
Physical activity (N/%) ^**e**^	Inactive	368 (19.2)	151 (16.9)
	Low	883 (46.1)	395 (44.2)
	Medium & High	660 (34.4)	344 (38.5)
Alcohol consumption (grams per day; mean ± SD)		10.3 ± 13.7	11.0 ± 14.4
Family history of cancer (N/%) ^**f**^	No	1004 (52.4)	468 (52.3)
	Yes	884 (46.1)	418 (46.8)
Cancer diagnosis (N/%)			
	Before BL	99 (5.2)	48 (5.4)
	Between BL and 8Y FU	158 (8.2)	62 (6.9)
	After 8Y FU	355 (18.5)	159 (17.8)
	Cancer free	1304 (68.1)	625 (69.9)

a Data missing for 1 participant in the total study population.

b Data missing for 42 and 21 participants in overall study population and individuals with repeated measurements.

c Data missing for 3 participants in overall study population.

d Data missing for 49 and 28 participants in overall study and individuals with repeated measurements.

e Data missing for 5 and 4 participants in overall study and individuals with repeated measurements.

f Data missing for 28 and 8 participants in overall study and individuals with repeated measurements.

BL, baseline; 8Y FU, 8-year follow-up.

### Association of PC-clock algorithms and cancer history in the overall study population

Given the persistent epigenetic changes associated with cancer initiation, progression, and related treatments^7,8,25^, we conducted multivariable linear regression analyses (Model 1 and Model 2, as described above) to examine the association between a cancer cases before baseline and BA measured at baseline (Supplementary Table 2). Participants with a cancer diagnosis prior to baseline had higher baseline BA than cancer-free participants for all five BA metrics with coefficient estimates (which represent adjusted differences in BA) ranging from 0.80 to 1.15. However, only PCHannum and PCGrimAge reached the significance threshold after Bonferroni-correction in the fully adjusted models (*p*-value < 0.01, Supplementary Table 2).

### Association of PC-clock algorithms and total cancer incidence in the overall study population

During over 21 years of follow-up, a total of 513 cancer cases were identified in the study population. Table 2 presents the associations of baseline DNAm-based PC-clock algorithms and cancer incidences in the overall study population. Multivariable-adjusted HRs for total incident cancer across all five PC-BAs ranged from 1.32 (95% CI per SD = 1.12 – 1.57) for PCHorvath to 1.50 (95% CI per SD = 1.17 – 1.91). Similar relationships of baseline PC-AgeAccels and total cancer incidence across all five PC-AgeAccel ranged from 1.17 (95% CI per SD = 1.06 - 1.28) for PCHorvathAgeAccel to 1.25 (95% CI per SD = 1.09 - 1.43) presented in Table 2.

**Table 2:** Association of baseline BA with total cancer risk

Predictors	Cancer free (N = 1304)	Incident cancer (N = 513)	Model 1		Model 2	
	Median (IQR)	Median (IQR)	HR per SD (95%CI)	*p*-value	HR per SD (95%CI)	*p*-value
**PC-biological age estimators**						
PCHorvath	65.56 (60.84 - 70.03)	67.70 (62.83 - 71.92)	1.36 (1.16 - 1.59)	**1.69E-4**	1.32 (1.12 - 1.57)	**0.001**
PCSkinBloodClock	62.29 (58.38 - 66.13)	63.63 (60.15 - 67.63)	1.36 (1.18 - 1.57)	**1.73E-5**	1.32 (1.14 - 1.53)	**2.89E-4**
PCHannum	66.22 (62.37 - 70.49)	67.80 (64.33 - 72.09)	1.42 (1.22 - 1.65)	**5.78E-6**	1.35 (1.15 - 1.58)	**2.59E-4**
PCPhenoAge	56.82 (52.37 - 61.42)	58.94 (53.65 - 63.25)	1.44 (1.23 - 1.69)	**4.93E-6**	1.38 (1.16 - 1.64)	**2.12E-4**
PCGrimAge	70.88 (66.77 - 75.20)	72.66 (68.85 - 76.67)	1.48 (1.25 - 1.76)	**7.73E-6**	1.50 (1.17 - 1.91)	**0.001**
**PC-biological age acceleration estimators**						
PCHorvathAgeAccel	-0.34 (-2.35 - 1.83)	0.10 (-1.83 - 2.22)	1.18 (1.08 - 1.29)	**1.69E-4**	1.17 (1.06 - 1.28)	**0.001**
PCSkinBloodClockAgeAccel	-0.27 (-2.47 - 1.89)	0.24 (-1.87 - 2.44)	1.21 (1.11 - 1.32)	**1.73E-5**	1.19 (1.08 - 1.31)	**2.89E-4**
PCHannumAgeAccel	-0.32 (-2.49 - 1.86)	0.16 (-1.66 - 2.32)	1.22 (1.12 - 1.33)	**5.78E-6**	1.18 (1.08 - 1.30)	**2.59E-4**
PCPhenoAgeAccel	-0.34 (-2.69 - 2.10)	0.28 (-2.36 - 2.87)	1.22 (1.12 - 1.33)	**4.93E-6**	1.19 (1.09 - 1.31)	**2.12E-4**
PCGrimAgeAccel	-0.69 (-2.41 - 1.56)	-0.20 (-1.94 - 2.47)	1.24 (1.13 - 1.37)	**7.73E-6**	1.25 (1.09 - 1.43)	**0.001**

Model 1: Cox proportional-hazard regression adjusted age, sex, blood cell composition and batch of measurements.

Model 2: additionally adjusting for BMI, smoking, and alcohol consumption, physical activity and family history of cancer.

The bold printed *p*-values passed Bonferroni-correction (0.05/5).

### Predictive value of BA for short- and long-term cancer risk

Based on the date of diagnosis, we stratified cancer outcomes into two groups: short-term events, defined as those diagnosed up to 8 years after baseline, and long-term events, defined as those diagnosed thereafter. Older baseline BA tended to be associated with higher short-term cancer risk (Table 3**and** Supplementary Table 3). In Model 1, PCSkinBloodClock and PCHannum were significantly associated with short-term cancer risk among all participants (HR per SD = 1.40, 95% CI 1.10 – 1.80, *p*-value = 0.007, and HR per SD = 1.52, 95%CI 1.16 – 1.99, *p*-value = 0.002, Supplementary Table 3). However, the associations were slightly attenuated by full adjustment and lost statistical significance after Bonferroni correction for multiple testing (Table 3).

**Table 3:** Association of baseline BA with cancer risk by diagnosis period

Predictors	Short-term incident cancer (N = 158)		Long-term incident cancer (N = 355)	
	HR per SD (95%CI)	*p*-value ^a^	HR per SD (95%CI)	*p*-value ^a^
PCHorvath	1.33 (0.98 - 1.80)	0.065	1.34 (1.09 - 1.65)	**0.005**
PCSkinBloodClock	1.32 (1.01 - 1.72)	0.045	1.35 (1.12 - 1.62)	**0.001**
PCHannum	1.41 (1.05 - 1.90)	0.021	1.36 (1.12 - 1.65)	**0.002**
PCPhenoAge	1.39 (1.01 - 1.91)	0.042	1.42 (1.15 - 1.74)	**9.21E-4**
PCGrimAge	1.23 (0.79 - 1.93)	0.354	1.67 (1.25 - 2.24)	**5.68E-4**

a Cox proportional-hazard regression adjusted for Model 2 variables.

The bold printed *p*-values passed Bonferroni-correction (0.05/5).

For the long-term risk analysis, the time scale started at the second blood draw for both cases and controls. Compared to short-term risk, BA exhibited a stronger predictive value for long-term cancer risk, as presented in Table 3**and** Supplementary Table 3. All baseline PC-clocks demonstrated significant associations with long-term cancer risk even after Bonferroni correction for multiple testing (HR per SD ranged from 1.34 to 1.67, *p*-value < 0.005, Table 3).

### Steeper BA trajectory indicates higher cancer risk

To explore the relationship of BA trajectory with cancer events after 8-year follow-up, we conducted Cox proportional-hazard regression models. The trajectory of BA was defined as the rate of biological aging per year calculated by the absolute change in BA divided by the individual time interval between baseline and 8-year follow-up. For the slope analysis, the time scale started at the second blood draw for both cases and controls, as the biological aging slope was defined using the two DNAm measurements. As shown in Table 4, four PC-clocks demonstrated relatively stable associations for overall cancer risk (HR per SD ranged from 1.33 for PCSkinBloodClock slope to 1.37 for PCHorvath slope in Model 2, *p*-value < 0.002).

**Table 4:** Association of BA slope with cancer incidence after 8-year follow-up

Predictors	Cancer free (N = 625)	Incident cancer (N = 159)	Model 1		Model 2	
	Median (IQR)	Median (IQR)	HR per SD (95%CI)	*p*-value	HR per SD (95%CI)	*p*-value
PCHorvath slope	0.52 (0.38 - 0.68)	0.59 (0.45 - 0.74)	1.29 (1.11 - 1.49)	**7.42E-4**	1.37 (1.16 - 1.61)	**1.86E-4**
PCSkinBloodClock slope	0.46 (0.27 - 0.65)	0.53 (0.36 - 0.69)	1.26 (1.07 - 1.48)	**0.005**	1.33 (1.12 - 1.59)	**0.001**
PCHannum slope	0.58 (0.35 - 0.78)	0.65 (0.48 - 0.85)	1.25 (1.06 - 1.48)	**0.008**	1.34 (1.11 - 1.61)	**0.002**
PCPhenoAge slope	0.56 (0.13 - 0.91)	0.63 (0.30 - 0.95)	1.26 (1.06 - 1.51)	**0.010**	1.34 (1.11 - 1.62)	**0.002**
PCGrimAge slope	0.61 (0.42 - 0.78)	0.64 (0.48 - 0.78)	1.17 (0.99 - 1.38)	0.072	1.24 (1.04 - 1.48)	0.018

Model 1: Cox proportional-hazard regression adjusted age, sex, blood cell composition and batch of measurements.

Model 2: additionally adjusting for BMI, smoking, and alcohol consumption, physical activity and family history of cancer.

The bold printed *p*-values passed Bonferroni-correction (0.05/5).

**Fig. 1:**
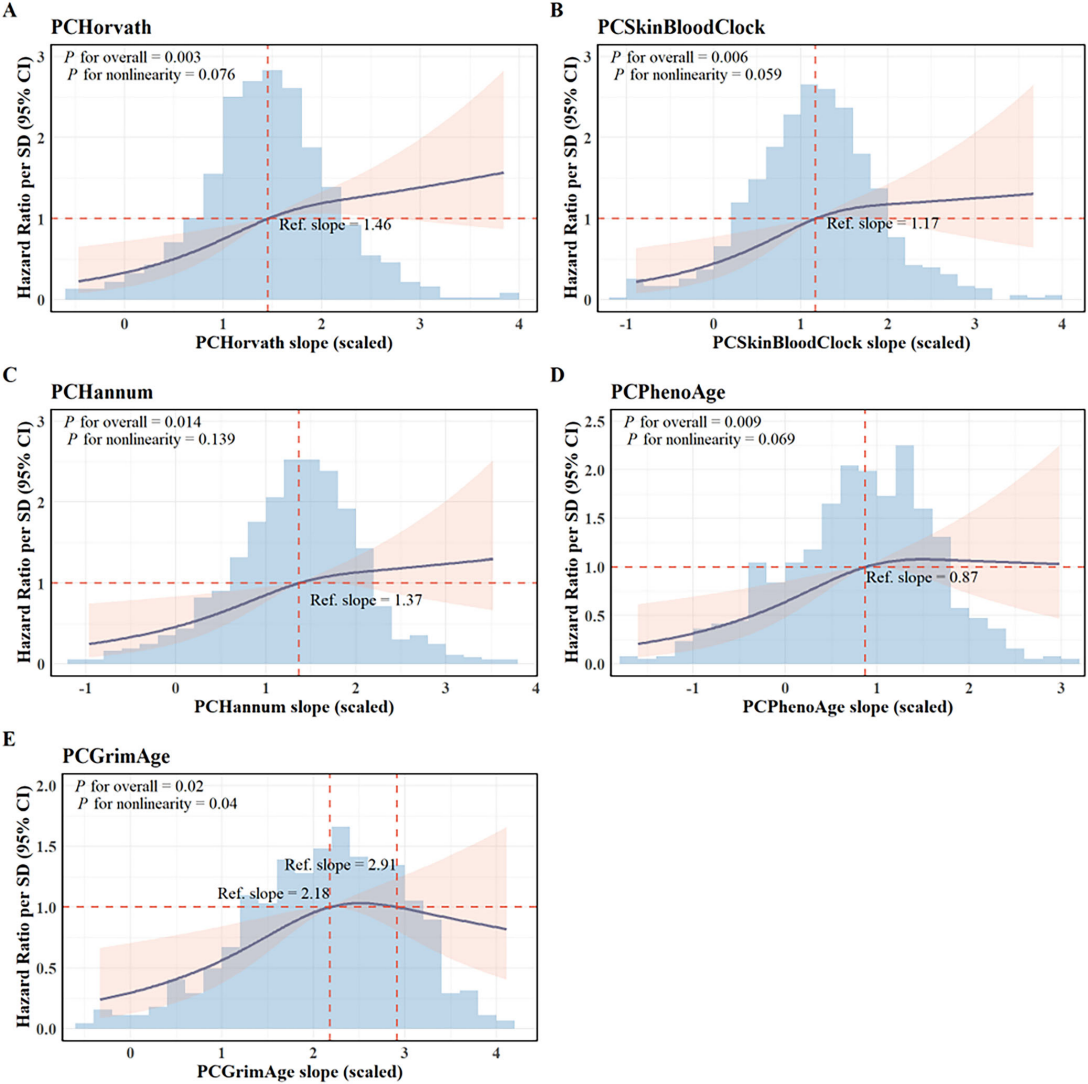
Dose-response relationships of PC-clock slopes with all type cancer risk. The models were adjusted for age, sex, nationality, leukocyte composition, education level, BMI, smoking status, alcohol consumption, BMI, physical activity and family history of cancer.

Restricted cubic spline analysis demonstrated a trend of increasing cancer risk with steeper trajectory of biological aging (Fig. 1). Even though the risk increase was steeper at lower trajectories of biological aging, the analysis did not provide statistically significant evidence of nonlinearity (*p* for nonlinearity > 0.05), except for the slope of PCGrimAge which showed an inverted U-shaped relationship with cancer risk (*p* for nonlinearity = 0.04). This spline analysis was exploratory, and the apparent nonlinearity at higher slope values may be influenced by the small number of participants in this range.

### Subgroup analyses

We replicated the analyses of the associations of PC-clocks slope with cancer incidence after 8-year follow-up in subgroups classified by sex, age group and family history of cancer (Supplementary Table 4 to 6). Increasing cancer risk was seen with increasing slope for all PC-clocks in both men and women, and the Bonferroni-corrected significance threshold was reached for three PC-clocks among men (PCHorvath, PCSkinBloodClock and PCHannum, Supplementary Table 4). When the models were separately fitted for age groups, stronger associations were seen for older participants (age > 60 years), with slopes in PCHorvath, PCHannum and PCPhenoAge reaching the Bonferroni-corrected significance threshold (Supplementary Table 5). Furthermore, stronger associations of PCHorvath, PCSkinBloodClock, and PCHannum slopes with cancer incidence were seen among participants without family history of cancer (reaching the Bonferroni-corrected significance threshold) than among those with (Supplementary Table 6).

## Discussion

This study investigated five epigenetic aging biomarkers (BA metrics) and their association with invasive cancer incidence in a population-based prospective longitudinal study of older adults with over 21 years follow-up. Older epigenetic age measured at both baseline and after 8-years of follow-up, as well as a steeper epigenetic aging trajectory was consistently associated with increased cancer risk, even though not all of the associations reached the strict Bonferroni-corrected threshold of statistical significance. Particularly strong positive associations were seen between BA slopes between baseline and 8-year follow-up and subsequent cancer risk, especially among older participants and those without a family history of cancer.

To our knowledge, this is the most comprehensive study on BA clocks, their longitudinal changes at older age and their relationship with cancer risk. The consistently positive associations which were observed and persisted after adjustment for CA and a variety of established cancer risk factors underline the intrinsic relationships between biological aging and increased cancer risk. Interestingly, these associations were strongest between BA trajectories and cancer risk at older ages. On the one hand, it is well known that the incidence of cancer increases substantially after the age of 60^8^. On the other hand, epigenetic events that are related to cancer risk are believed to occur in the early process of cancer development^26^. With the constant effects of the epigenetic events on genomic stability and gene expression, these changes might result in carcinogenesis from initiation through progression^5,7,8^. Especially, in later life, the homeostatic mechanisms responsible for maintaining biochemical balance and preventing functional decline may become disrupted, leading to an amplification of aging-related differences over time^9^.

Despite the consistent positive associations between BA and BA trajectories and cancer risk seen in our study, the observational nature of our study precludes drawing causal inferences. Nevertheless, our results indicate that BA clocks and BA trajectories are strongly associated with cancer risk and may provide complementary information beyond chronological age, one of the strongest risk factors for cancer. Although there is considerable heterogeneity in the pace and pattern of aging across the life course^27^, we adopted a longitudinal framework that conceptualizes cancer as a chronic disease arising from cumulative biological aging processes^6,9^. While most individuals experience biologically “normal” aging, some exhibit accelerated aging, i.e., aging more rapidly than the norm, due to genetic predisposition, lifestyle factors, or yet undiscovered factors^28^. In our study, we detected varying degrees of accelerated aging in both participants diagnosed with cancer prior to baseline and those who developed cancer during follow-up. This acceleration may have originated early or late in life, rendering these individuals more susceptible to cancer. Survivors diagnosed with cancer in midlife or later life represent a heterogeneous group, each already on a distinct trajectory of biological aging within and across CA. While DunedinPACE provides an estimate of the pace of biological aging at a single time point, the present study focused on longitudinal aging trajectories captured by changes in DNAm-based biological age over time, which reflect the average aging rate across the follow-up interval.

In the interpretation of our study, a number of specific strengths and limitations deserve careful consideration. Strengths include that it is based on a large population-based cohort study with extensive collection of biospecimen, life style and medical data, and comprehensive long-term prospective follow-up with respect to morbidity and mortality. In particular, record linkage with the Saarland Cancer Registry ensured complete ascertainment of outcomes. Additionally, the longitudinal design allowed us to assess BA average trajectories over an 8-year period and link them to subsequent 12-year cancer-related outcomes. Nevertheless, despite the overall large sample size, a substantial proportion of the observed, consistently positive associations between BA clocks and BA trajectories did not reach the strict Bonferroni-corrected level of significance. Furthermore, due to sample size limitations, only total cancer risk could be assessed with reasonable power and precision. Preliminary studies based on BA measurements at a single point of time, including a previous analysis from the ESTHER study, have suggested that such associations may substantially vary by cancer type^29^. Further, much larger studies with longitudinal BA measurements are required to assess predictive value for the risk of specific cancers. Such studies may also help to define a potential role of BA clocks and BA trajectories in novel approaches of risk-adapted cancer screening. Despite the strength of including BA trajectories as a predictor, measurement of BA trajectories requires survival of participants between baseline and follow-up measurements which may imply survival bias if baseline BA is strongly related to total mortality. Nevertheless, although associations with total mortality have been reported, major survival bias seems unlikely in our study, given limited overall mortality in the ESTHER cohort up to 8 years after baseline.

In conclusion, despite its limitations, our study demonstrates strong associations between BA, particularly accelerated BA, and total cancer risk in older adults, independent of established cancer risk factors such as chronological age, sex, and major lifestyle factors. Further research, preferably based on large-scale cohorts with multiple longitudinal BA measurements, should aim for defining a potential role of BA monitoring for clinical practice, such as primary, secondary or tertiary prevention of overall cancer or specific types of cancer.

## Methods

### Study population and data collection

The study population was drawn from the ESTHER cohort, an ongoing population-based cohort study conducted in Saarland, Germany. Details of the study design have been described previously^6,30–32^. Briefly, 9,940 individuals aged 50–75 years were recruited by their general practitioners (GPs) during routine health screenings between July 2000 and December 2002, and were followed up every two to three years thereafter. At baseline and each follow-up, standardized self-administered questionnaires were used to collect information on sociodemographic characteristics, lifestyle, and dietary habits. General health examination results were documented by GPs using standardized forms. Blood samples were collected during examinations and stored at −80 °C for future analyses. Cancer incidence and mortality follow-up was conducted by record linkage with cancer and population registries. The ESTHER study adhered to the principles of the Declaration of Helsinki and was approved by the ethics committees of the medical faculty of the University of Heidelberg (Application number: S-58/2000) and the medical board of the state of Saarland. Written informed consent was obtained from each participant.

The ESTHER population has been shown to be representative of the general German population of the same age group with respect to key sociodemographic variables and risk factor profiles^33^. For this study, a random sample of 1930 participants were selected from the ESTHER study population for epigenome-wide blood DNAm measurements at baseline that were carried out in two different batches. A subsample of 900 participants also had epigenome-wide blood DNAm measurements at 8-year follow-up.

### Methylation assessment

DNA from whole blood samples obtained at recruitment was extracted using a salting-out procedure^34^ and paired samples from the same individual taken at 8-year follow-up were processed on the same array to minimize technical variation. Genome-wide DNAm was assessed using the Infinium MethylationEPIC (850 K) BeadChip Kit (Illumina, San Diego, CA, USA), according to the manufacturer’s instructions, by the Genomics and Proteomics Core Facility at the German Cancer Research Center (DKFZ), Heidelberg, Germany^30,35,36^. During preprocessing, probes with detection p-values > 0.01, those with >10% missing values, and probes targeting the X or Y chromosomes were excluded^30,35,36^. After quality control and exclusion of low-quality samples, a total of 1916 participants were retained for subsequent analyses.

### Sample exclusions

**Fig. 2:**
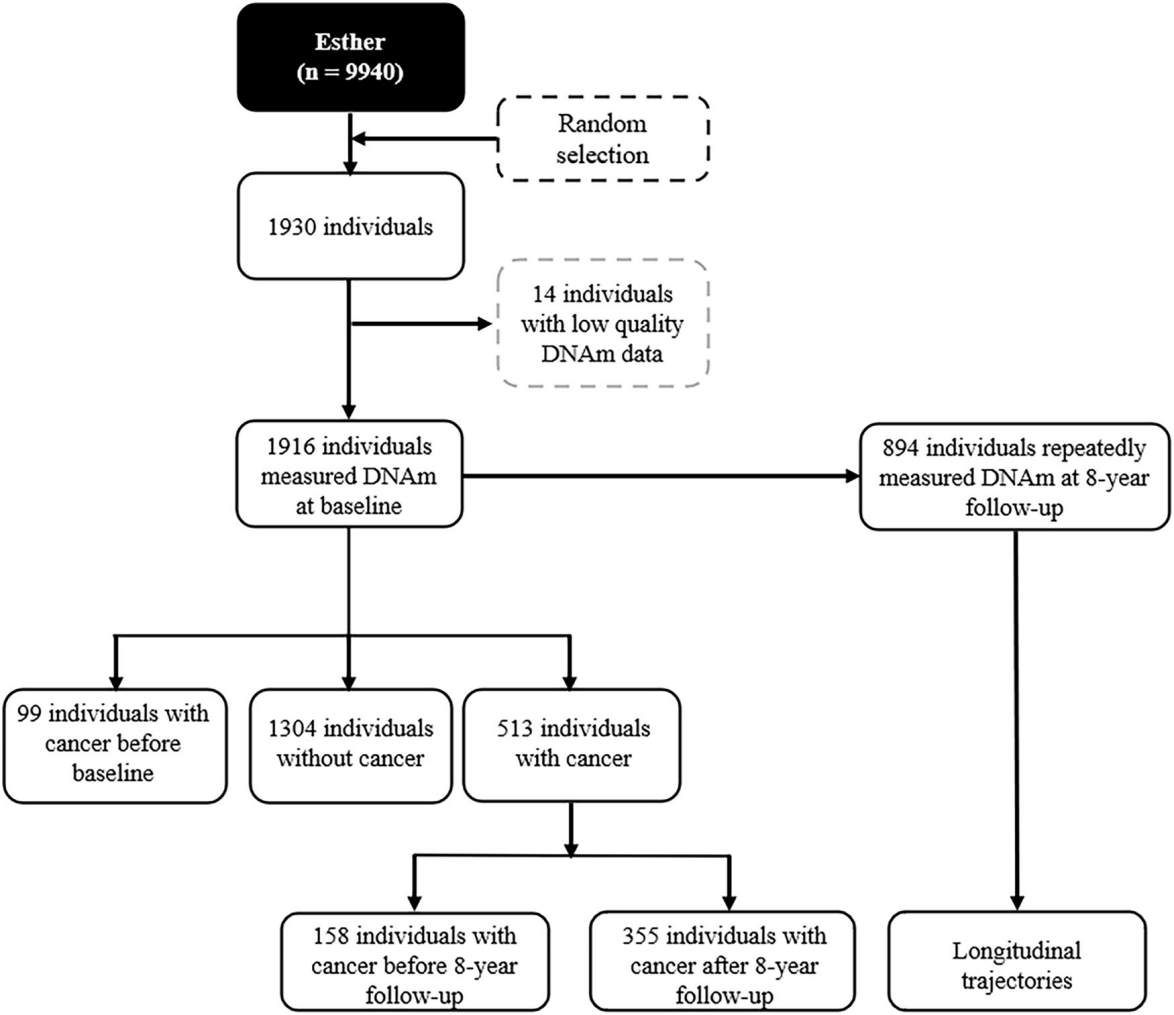
Study participant flow chat. The ESTHER cohort at baseline contained a total of 9940 individuals. 1930 individuals were randomly selected for DNA methylation testing in baseline blood samples. Of these, 14 individuals lacking high-quality methylation results were excluded, leaving 1916 individuals. Ninety-nine individuals were diagnosed with cancer prior to the baseline, and 513 were diagnosed with cancer after baseline, of whom 355 were diagnosed after 8-year follow-up.

Figure 2 presents a flow chart of the selection of the study population. Initially, 1930 participants were randomly selected for DNAm analyses. After excluding 14 participants with low-quality DNAm data, 1916 individuals were included, of whom 894 also had DNAm measured at 8-year follow-up. Ninety-nine participants had a cancer diagnosis before baseline, and 513 incident cancer cases were observed during a median follow-up of 20.6 years (IQR: 14.4 – 21.5 years), of which 158 and 355 cases were diagnosed between baseline and 8-year follow-up, and after 8-year follow-up, respectively.

### Estimation of epigenetic age metrics

Five PC-based epigenetic clocks were calculated in this study, including PC-Horvath Pan-Tissue (PCHorvath), PC-Skin & Blood Clock (PCSkinBloodClock), PC-Hannum DNAmAge (PCHannum), PC-PhenoAge (PCPhenoAge) and PC-GrimAge (PCGrimAge). PC clocks were calculated using the R script and 78,464 CpG sites per sample, as described in a previous report^24^. Missing CpG values (less than 5%) were imputed using mean substitution. Age acceleration (PCAgeAccel) was defined as the residual from regressing DNAm age estimates on CA^16^, with regressions performed separately for baseline and follow-up measurements. Separate residualization was used to accommodate the longitudinal design with repeated measures and to avoid treating multiple observations from the same individual as independent. For individuals with DNAm data measured at both baseline and 8-year follow-up, individuals BA slope of linear regression was interpreted as the average rate of biological aging per year, calculated as the absolute changes in DNAm age over the time period divided by the time interval between baseline and 8-year follow-up. If the PC clock slope is > 0, it indicates an accelerated rate of epigenetic age change.

### Ascertainment of cancer cases

Incident cases of cancer, including total cancer (ICD-10 codes C00–C97 excluding the code C44 for non-melanoma skin cancer) diagnosed between 2000 and end of 2022 were identified through record linkage with the Saarland Cancer Registry. The history of invasive tumors before baseline was determined by either self-report or record linkage with data from the Saarland Cancer Registry, which started to record cancers conditions in 1970. The distribution of cancer sites among prevalent and incident cases is summarized in Supplementary Table 1.

### Covariates assessment

Several covariates were considered, including information on sociodemographic characteristics, smoking status, physical activity and alcohol consumption, which were obtained from a standardized self-administered questionnaire. Height and weight were assessed and documented on a standardized form by GPs during the health examination and body mass index (BMI) was calculated as weight in kilograms divided by height in meters squared (kg/m²) and categorized according to the World Health Organization classification.

### Statistical methods

Standard descriptive methods were used to describe demographic characteristics of the study subjects at baseline. Multivariable linear regression was used to examine the association of a previous cancer diagnosis with BA at baseline. The models were firstly adjusted for age, sex, leukocyte composition (estimated by the Houseman approach)^37^, and batch (Model 1), and we additionally controlled for educational level ( ≤ 9 years, 10-11 years, and ≥12 years), smoking status (never smoker, former smoker, current smoker), alcohol consumption (grams per day), BMI (kg/m2), physical activity (inactive, low, medium, or high) and family history of cancer (yes/no) (Model 2).

To estimate the predictive value of BA and BA slope on cancer incidence, we performed Cox proportional hazard models with the same adjustments of Model 1 and Model 2. The proportional hazards assumption was checked by scaled Schoenfeld residuals plots^38^. Hazard ratios (HR) with corresponding 95% confidence intervals (CI) per standard deviation (SD) increase in BA and BA slope were calculated for cancer risk in the entire study population and separately for individuals with repeated DNAm data. Cox proportional hazards models were conducted using time-on-study as the time scale. Follow-up time was defined as age at event or censoring minus age at baseline. Baseline age was included as a covariate in models of baseline epigenetic aging measures, whereas age at the second blood draw was used as the age covariate in slope analyses, reflecting the time point at which the aging slope was defined.

To evaluate the exposure-response relationships between slope of BA and overall cancer incidence, restricted cubic spline (RCS) models with three knots at 25th, 50th and 75th percentiles were applied. We generated scatter plots to evaluate the relationship between the PC-clocks trajectories and the time to cancer diagnosis among the individuals with two-wave methylation data. Furthermore, we conducted subgroup specific analyses for the associations of the BA slope with incident cancer by sex, age ( ≤ 60 years / > 60 years) and family history of cancer (without / with) in individuals with multiple measurements of DNAm. For age-stratified slope analyses, participants were stratified according to chronological age at baseline. Baseline age was used to define age strata to ensure stable group assignment throughout the longitudinal assessment period.

All statistical analyses were carried out using the R software environment for statistical computing (version 3.6.0, Vienna, Austria). Bonferroni-correction was performed to reduce the likelihood of type Ⅰ statistical error, and statistical significance was defined by *p*-value < 0.05 in two-sided testing ( < 0.05/5 = 0.01 after Bonferroni correction for five different BA metrics).

## Supplementary information

Supplementary_revised.

## Data Availability

The data that support the findings of this study are not openly available due to reasons of sensitivity.
