# Supplementary material for: Epigenetic aging and cancer incidence in a German cohort of older adults
Source: NPJ Aging. 2026 Mar 9;12(1):41. doi: 10.1038/s41514-026-00356-y (PMC12992613; doi:10.1038/s41514-026-00356-y)
Supplement: Supplementary file 1 — Supplementary_revised. [file 41514_2026_356_MOESM1_ESM.docx]

**Supplementary Tables**

**Supplementary Table 1. Distribution of cancer sites among prevalent and incident cancer cases during follow-up**

|  |  | Total study population (N = 612) | | Individuals with repeated measurement (N = 269) | |
| --- | --- | --- | --- | --- | --- |
|  |  | Prevalent (N = 99) | Incident (N = 513) | Prevalent (N = 48) | Incident (N = 221) |
| Cancer types (N/%) | Breast Cancer | 14 (14.1) | 74 (14.4) | 5 (10.4) | 39 (17.6) |
|  | Lung Cancer | 5 (5.1) | 54 (10.5) | 3 (6.3) | 19 (8.6) |
|  | Prostate Cancer | 10 (10.1) | 109 (21.2) | 6 (12.5) | 44 (19.9) |
|  | Colon Cancer | 12 (12.1) | 40 (7.8) | 10 (20.8) | 15 (6.8) |
|  | Rectal Cancer | 11 (11.1) | 27 (5.3) | 8 (16.7) | 12 (5.4) |
|  | Other | 47 (47.5) | 209 (40.7) | 16 (33.3) | 92 (41.6) |

**Supplementary Table 2.** Association of cancer diagnosed before baseline with baseline BA

| Outcomes | Cancer Free  (N=1304) | Cancer before BL (N=99) | Model 1 |  | Model 2 |  |
| --- | --- | --- | --- | --- | --- | --- |
|  | Median (IQR) | Median (IQR) | β (95%CI) | *p*-value | β (95%CI) | *p*-value |
| PCHorvath | 65.56 (60.84 - 70.03) | 68.79 (64.57 - 72.33) | 0.93 (0.21 - 1.65) | 0.011 | 0.98 (0.22 - 1.74) | 0.012 |
| PCSkinBloodClock | 62.29 (58.38 - 66.13) | 64.70 (61.64 - 69.73) | 0.87 (0.15 - 1.59) | 0.018 | 0.85 (0.09 - 1.61) | 0.028 |
| PCHannum | 66.22 (62.37 - 70.49) | 70.40 (65.64 - 74.67) | 1.03 (0.35 - 1.71) | **0.003** | 1.15 (0.42 - 1.88) | **0.002** |
| PCPhenoAge | 56.82 (52.37 - 61.42) | 60.55 (55.10 - 65.60) | 0.96 (0.19 - 1.73) | 0.015 | 1.04 (0.23 - 1.84) | 0.011 |
| PCGrimAge | 70.88 (66.77 - 75.20) | 74.08 (70.05 - 77.73) | 0.55 (-0.03 - 1.13) | 0.064 | 0.80 (0.32 - 1.29) | **0.001** |
| Model 1: adjusting for age, sex, blood cell composition and batch of measurements  Model 2: additionally adjusting for BMI, smoking, and alcohol consumption, physical activity and family history of cancer  The bold printed *p*-values passed Bonferroni-correction (0.05/5) | | | | | | |

**Supplementary Table 3**: Association of baseline BA with cancer risk by diagnosis period

|  | Cancer Free | Incident cancer | Model 1 |  | Model 2 |  |
| --- | --- | --- | --- | --- | --- | --- |
|  | Median (IQR) | Median (IQR) | HR per SD (95%CI) | *p*-value | HR per SD (95%CI) | *p*-value |
| 1304 cancer free vs.158 short-term incident cancer: | | | | | | |
| PCHorvath | 65.56 (60.84 - 70.03) | 68.73 (62.87 - 73.31) | 1.43 (1.08 - 1.88) | 0.011 | 1.33 (0.98 - 1.80) | 0.065 |
| PCSkinBloodClock | 62.29 (58.38 - 66.13) | 64.03 (60.27 - 67.90) | 1.40 (1.10 - 1.80) | **0.007** | 1.32 (1.01 - 1.72) | 0.045 |
| PCHannum | 66.22 (62.37 - 70.49) | 69.02 (65.25 - 72.74) | 1.52 (1.16 - 1.99) | **0.002** | 1.41 (1.05 - 1.90) | 0.021 |
| PCPhenoAge | 56.82 (52.37 - 61.42) | 59.44 (54.66 - 63.60) | 1.41 (1.06 - 1.89) | 0.019 | 1.39 (1.01 - 1.91) | 0.042 |
| PCGrimAge | 70.88 (66.77 - 75.20) | 73.22 (69.08 - 77.58) | 1.36 (0.99 - 1.87) | 0.055 | 1.23 (0.79 - 1.93) | 0.354 |
| 1304 cancer free vs.355 long-term incident cancer | | | | | | |
| PCHorvath | 65.56 (60.84 - 70.03) | 67.22 (62.78 - 71.30) | 1.35 (1.11 - 1.64) | **0.003** | 1.34 (1.09 - 1.65) | **0.005** |
| PCSkinBloodClock | 62.29 (58.38 - 66.13) | 63.48 (60.10 - 67.41) | 1.37 (1.15 - 1.62) | **3.39E-4** | 1.35 (1.12 - 1.62) | **0.001** |
| PCHannum | 66.22 (62.37 - 70.49) | 67.55 (63.74 - 71.83) | 1.41 (1.17 - 1.69) | **2.48E-4** | 1.36 (1.12 - 1.65) | **0.002** |
| PCPhenoAge | 56.82 (52.37 - 61.42) | 58.70 (53.20 - 63.11) | 1.51 (1.25 - 1.83) | **1.92E-5** | 1.42 (1.15 - 1.74) | **9.21E-4** |
| PCGrimAge | 70.88 (66.77 - 75.20) | 72.51 (68.81 - 76.13) | 1.58 (1.28 - 1.94) | **1.78E-5** | 1.67 (1.25 - 2.24) | **5.68E-4** |
| Model 1: Cox proportional-hazard regression adjusted age, sex, blood cell composition and batch of measurements  Model 2: additionally adjusting for BMI, smoking, and alcohol consumption, physical activity and family history of cancer  The bold printed *p*-values passed Bonferroni-correction (0.05/5) | | | | | | |

**Supplementary Table 4.** Association of BA slope with cancer incidence after 8-year follow-up, stratified by sex

| Predictors | Male  (N = 408) | | Female  (N = 486) | |
| --- | --- | --- | --- | --- |
|  | HR per SD (95%CI) | *p*-value | HR per SD (95%CI) | *p*-value |
| PCHorvath slope | 1.33 (1.08 - 1.62) | **0.006** | 1.53 (1.09 - 2.14) | 0.015 |
| PCSkinBloodClock slope | 1.39 (1.11 - 1.72) | **0.004** | 1.24 (0.93 - 1.67) | 0.141 |
| PCHannum slope | 1.42 (1.11 - 1.81) | **0.006** | 1.28 (0.96 - 1.71) | 0.088 |
| PCPhenoAge slope | 1.31 (1.02 - 1.67) | 0.032 | 1.40 (1.03 - 1.90) | 0.032 |
| PCGrimAge slope | 1.16 (0.93 - 1.45) | 0.185 | 1.44 (1.05 - 1.98) | 0.024 |
| HR, Hazard Ratio; SD, standard deviation; CI, Confidence Interval.  The table shows the results of Model 2, adjusted for age, sex, blood cell composition and batch of measurements, BMI, smoking, and alcohol consumption, physical activity and family history of cancer. The bold printed *p*-values passed Bonferroni-correction (0.05/5). | | | | |

**Supplementary Table 5.** Association of BA slope with long-term cancer incidence, stratified by age

| Predictors | Age ≤ 60  (N = 378) | | Age > 60  (N = 516) | |
| --- | --- | --- | --- | --- |
|  | HR per SD (95%CI) | *p*-value | HR per SD (95%CI) | *p*-value |
| PCHorvath slope | 1.29 (1.05 - 1.58) | 0.016 | 1.77 (1.21 - 2.58) | **0.003** |
| PCSkinBloodClock slope | 1.29 (1.04 - 1.61) | 0.023 | 1.51 (1.06 - 2.15) | 0.021 |
| PCHannum slope | 1.21 (0.95 - 1.54) | 0.124 | 1.70 (1.17 - 2.45) | **0.005** |
| PCPhenoAge slope | 1.18 (0.92 - 1.51) | 0.195 | 1.80 (1.26 - 2.56) | **0.001** |
| PCGrimAge slope | 1.19 (0.93 - 1.52) | 0.167 | 1.37 (1.03 - 1.83) | 0.030 |
| HR, Hazard Ratio; SD, standard deviation; CI, Confidence Interval.  The table shows the results of Model 2, adjusted for age, sex, blood cell composition and batch of measurements, BMI, smoking, and alcohol consumption, physical activity and family history of cancer. The bold printed *p*-values passed Bonferroni-correction (0.05/5). | | | | |

**Supplementary Table 6.** Association of BA slope with long-term cancer incidence, stratified by family history

| Predictors | Without family history  (N = 468) | | With family history  (N = 418) | |
| --- | --- | --- | --- | --- |
|  | HR per SD (95%CI) | *p*-value | HR per SD (95%CI) | *p*-value |
| PCHorvath slope | 1.52 (1.16 - 1.99) | **0.002** | 1.30 (1.02 - 1.64) | 0.032 |
| PCSkinBloodClock slope | 1.47 (1.10 - 1.96) | **0.009** | 1.26 (0.99 - 1.61) | 0.059 |
| PCHannum slope | 1.57 (1.14 - 2.15) | **0.005** | 1.23 (0.96 - 1.59) | 0.105 |
| PCPhenoAge slope | 1.34 (1.00 - 1.79) | 0.049 | 1.35 (1.05 - 1.75) | 0.020 |
| PCGrimAge slope | 1.13 (0.88 - 1.46) | 0.331 | 1.36 (1.05 - 1.75) | 0.020 |
| HR, Hazard Ratio; SD, standard deviation; CI, Confidence Interval.  The table shows the results of Model 2, adjusted for age, sex, blood cell composition and batch of measurements, BMI, smoking, and alcohol consumption, physical activity and family history of cancer. The bold printed *p*-values passed Bonferroni-correction (0.05/5). | | | | |
